# Diffuse Optical Characterization of the Healthy Human Thyroid Tissue and Two Pathological Case Studies

**DOI:** 10.1371/journal.pone.0147851

**Published:** 2016-01-27

**Authors:** Claus Lindner, Mireia Mora, Parisa Farzam, Mattia Squarcia, Johannes Johansson, Udo M. Weigel, Irene Halperin, Felicia A. Hanzu, Turgut Durduran

**Affiliations:** 1 ICFO - Institut de Ciències Fotòniques, The Barcelona Institute of Science and Technology, Castelldefels (Barcelona), Spain; 2 Department of Endocrinology and Nutrition, Hospital Clínic, Barcelona, Spain; 3 Institut d’Investigacions Biomèdiques August Pi I Sunyer (IDIBAPS), Barcelona, Spain; 4 Department of Radiology, Hospital Clínic, Barcelona, Spain; 5 Hemophotonics S.L., Mediterranean Technology Park, Castelldefels (Barcelona), Spain; 6 Institució Catalana de Recerca i Estudis Avançats (ICREA), Barcelona, Spain; Tufts University, UNITED STATES

## Abstract

The *in vivo* optical and hemodynamic properties of the healthy (n = 22) and pathological (n = 2) human thyroid tissue were measured non-invasively using a custom time-resolved spectroscopy (TRS) and diffuse correlation spectroscopy (DCS) system. Medical ultrasound was used to guide the placement of the hand-held hybrid optical probe. TRS measured the absorption and reduced scattering coefficients (*μ*_a_, *μ*_s_′) at three wavelengths (690, 785 and 830 nm) to derive total hemoglobin concentration (THC) and oxygen saturation (StO_2_). DCS measured the microvascular blood flow index (BFI). Their dependencies on physiological and clinical parameters and positions along the thyroid were investigated and compared to the surrounding sternocleidomastoid muscle. The THC in the thyroid ranged from 131.9 *μ*M to 144.8 *μ*M, showing a 25–44% increase compared to the surrounding sternocleidomastoid muscle tissue. The blood flow was significantly higher in the thyroid (BFI_thyroid_ = 16.0 × 10^-9^ cm^2^/s) compared to the muscle (BFI_muscle_ = 7.8 × 10^-9^ cm^2^/s), while StO_2_ showed a small (StO_2, muscle_ = 63.8% to StO_2, thyroid_ = 68.4%), yet significant difference. Two case studies with thyroid nodules underwent the same measurement protocol prior to thyroidectomy. Their THC and BFI reached values around 226.5 *μ*M and 62.8 × 10^-9^ cm^2^/s respectively showing a clear contrast to the nodule-free thyroid tissue as well as the general population. The initial characterization of the healthy and pathologic human thyroid tissue lays the ground work for the future investigation on the use of diffuse optics in thyroid cancer screening.

## Introduction

Thyroid nodules (TN) vary in size and are a common pathology in endocrinology. The statistics show that palpable nodules have a prevalence around 5% in women and 1% in men. These numbers increase to 19–76% [1, 2] when neck ultrasound (US) is used for screening in addition to palpation. Approximately 5–15% of these nodules turn out to be thyroid cancer (TC) [1] which is the most common malignant tumor of the endocrine system. The incidence of thyroid cancer is increasing more rapidly than any other cancer type in the modern world [3].

With exception of the relatively rare poorly differentiated and anaplastic forms, TC prognosis is generally good. The 10-year survival rates—corrected for age and sex—range from 95% for papillary to 90% (follicular) and down to 13% for the anaplastic TC [4]. While TC has a low overall mortality, in specific cases of low conventional treatment response, aggressive behavior, persistence or recurrence of the disease and no alternative treatment option, the mortality rate is substantially higher.

The initial precise characterization and diagnosis of the thyroid nodule is critical in order to decide on the course of action. The options range from surgical resection to follow-up or—in the case of presumed TC—an extensive initial surgical procedure. Unfortunately, the current sensitivity and specificity of the first point-of-care, non-invasive screening method, ultrasound, is quite poor, ranging from 17–87% in sensitivity and from 39–95% in specificity depending on the type of the malignant nodules [3, 5–8]. Unfortunately, even the ultrasound-guided fine needle aspiration biopsy (FNAB)—which follows a positive ultrasound screening result—of the suspicious nodule is also limited in sensitivity and specificity [1].

In addition to various other parameters (e.g. elastography), it has been speculated that the inclusion of the nodule hemodynamics, in particular that of microvascular blood flow, may improve the screening process. In fact, recent studies have observed that papillary carcinomas are richer in microvessels (95.75%) than adenomas (49%) and capillaries are more frequent in adenomas (9%) than in carcinomas (1%) [9]. However, ultrasound has limited sensitivity to microvascular blood flow, and, therefore, complementary modalities are being sought.

In short, the high prevalence of thyroid nodules and the above-mentioned limitations of the existing screening strategies call for improvements in strategies to identify lesions. If successful, these may have an immense socio-economic impact reducing unnecessary FNABs or surgeries and improving the response to therapies.

Several techniques, such as (time-resolved) fluorescence [10–13], Raman [14, 15], elastic scattering (ESS) [16] spectroscopy and two-photon excited fluorescence (TPEF) [17] in combination with second-harmonic generation (SHG) [18] using light have been suggested for improving the cancer and, in particular, the thyroid cancer screening process. However, these are invasive techniques focusing on different aspects of light interaction with tissue. ESS, TPEF and SHG suggest an improvement of the thyroid screening process by focusing on the cellular tissue morphology which influences the spatial variations in the refractive index, which in turn result in alterations of photon scattering. Overall, these methods suggest that there is an optical contrast between the healthy and pathologic thyroid tissue.

In fact, the light scattering characteristics of tissues together with its light absorption spectrum is also used in the near-infrared diffuse optical spectroscopy (DOS). Therefore, we hypothesize that since diffuse optical methods are sensitive to the hemodynamics of the microvasculature and tissue scattering, they may play an important role in the screening and characterization of thyroid nodules for malignancy as well as the overall thyroid characterization [19–21]. DOS allows the non-invasive characterization of the wavelength dependent absorption and scattering properties of tissues from which the microvascular total hemoglobin concentration (THC), blood oxygen saturation (StO_2_) and the concentration of water, lipids and other tissue chromophores can be derived. It has been widely applied in characterizing tumor tissues, specially in optical mammography, and for the monitoring of the local tissue response to therapy [22–24]. Diffuse correlation spectroscopy (DCS) [19, 25, 26] is another related technique which allows the direct measurement of the deep local microvascular blood flow [27]. DCS has also been applied to the characterization of tumors and to monitor the therapy response [20, 21, 28–31].

**Fig 1:**
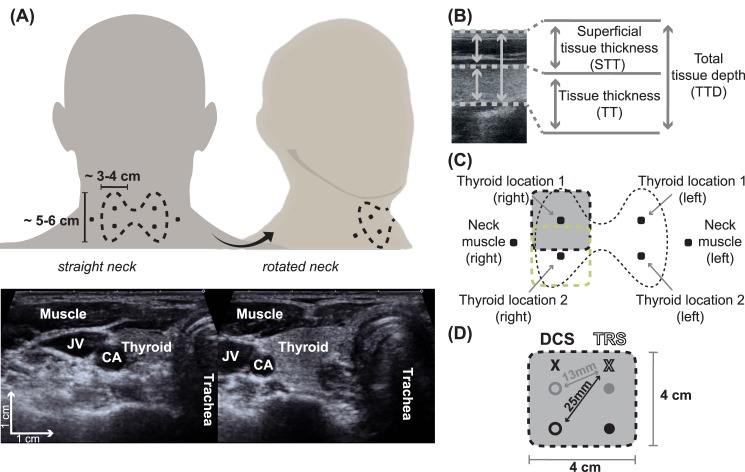
Protocol schematics with measurement locations, tissue dimensions and probe geometry. Panel (A) Shows the location of the thyroid, its dimensions and the defined measurement points for this study together with a head turn used to expose the thyroid. The ultrasound images illustrate the locations of the jugular vein (JV), carotid artery (CA), esophagus (EPG), the (left) thyroid gland, its position and superficial structures (sternocleidomastoid muscle, skin and adipose layers) in normal neck (right) and rotated neck (left) position; Panel (B) shows an excerpt of an ultrasound image from a thyroid gland. The total tissue depth (TTD) is defined by the sum of the superficial tissues (STT) and the tissue thickness (TT). Typical values vary between different tissue types; Panel (C) illustrates the three different measurement points per side. The probe size and its placement are shown in relation to the thyroid gland size; Panel (D) shows the hybrid diffuse optics probe which consisted of one source per modality (DCS and TRS) and two different detector locations per source. For a good overlap for the probed regions by both modalities, we have used a cross-geometry with the DCS source on the left side, its detectors on the right side of the probe and for TRS vice-versa. The shorter source-detector separation (SD) was used in a subset of eleven subjects, which is half of the study population, as well as the two pathology cases.

To the best of our knowledge, DOS or DCS have not yet been utilized to characterize the thyroid gland or its pathophysiology. Here we hypothesize that the thyroid gland is accessible to the non-invasive, hand-held DOS/DCS hybrid optical probes (see Fig 1(A) and 1(B) for the illustration of the thyroid gland and its anatomy) and that—in healthy condition—it is relatively homogeneous. Moreover, due to the increased microvasculature in TN [9], they are expected to present a measurable contrast, facilitating in the end the use of diffuse optics as an additional tool to improve the limited contrast of the current standard screening process [1, 3, 5–8]. The ultimate goal is to help classify the nodules as benign or malignant. This study aims to test the first part of this hypothesis by characterizing the homogeneity of the thyroid gland and compare the results to the adjacent neck muscles (sternocleidomastoid) using a custom-made time-resolved spectroscopy (TRS) and DCS hybrid instrument and probe [32]. Furthermore, we present two case studies with different thyroid pathologies to begin to address the second part of the hypothesis. Further studies by diffuse optical data on thyroid nodules should enable us to establish ranges and relationships between different measurable parameters, indicating the probability of malignancy.

The measurements were guided by ultrasound imaging and its assessment by expert radiologists and endocrinologists. We have quantified the depth and the extent of the thyroid gland and investigated our results to look for correlations between different physical, demographic and clinical parameters. This approach enabled us to establish a range of typical healthy thyroid tissue parameters to serve as a reference for future measurements on thyroid pathologies thus paving the way towards exploring the utility of this method for improved thyroid screening.

## Methods

### Protocol

All procedures and devices of this study were approved by the ethical committee of Hospital Clínic in Barcelona (CEIC—Comité Ético de Investigación Clínica del Hospital Clínic de Barcelona). Each subject signed an informed consent and the study has been conducted according to the principles of the Declaration of Helsinki. The volunteers were screened by ultrasound to evaluate the thyroid condition. The presence of diffuse thyroid diseases, such as thyroiditis, hyper- or hypothyroidism and Graves’ disease, as well as benign and/or malign nodules and cervical adenopathies were the main exclusion criteria for the healthy population.

Each subject’s tissue dimensions in the marked probe locations (see Fig 1(A)) were extracted from the ultrasound images. We have defined three representative parameters (see Fig 1(B)): the total tissue depth (TTD), superficial tissue thickness (STT) and tissue thickness (TT). The latter is given by the depth extension of the tissue of interest, i.e., muscle or thyroid tissue. This tissue thickness and the superficial tissues (skin and adipose layers) add up to the total tissue depth, which is defined by total distance from the skin surface to the end of the thyroid or muscle tissue.

In order to evaluate the general health condition of subjects, they underwent a basic health screening, which consisted of questions about hypertension, diabetes, hyperlipidemia, arterial fibrillation, congestive heart failure, coronary artery disease, previous myocardial infarction, carotid artery disease, smoking, lung and/or kidney diseases and the list of current medications.

Furthermore, we have noted age, height, weight, body mass index (BMI), heart rate (HR) and arterial oxygen saturation (SaO_2_) before the diffuse optical measurement as well as the systolic and diastolic blood pressure (BP_sys_, BP_dia_) before and after the data recording procedure. These serve as a check for the subject’s general well-being in addition to the above-mentioned basic health screening. Moreover, the blood pressure reading prior and after the measurement procedure provide further information about the global hemodynamic condition over the course of the experiment.

The measurement protocol consisted of several probe placements on the thyroid and the neck sternocleidomastoid muscle as a reference point as illustrated in Fig 1(A) and 1(C).

The subjects were laid down with a slight head tilt backwards to expose the thyroid. Furthermore, we have used ultrasound to explore the optimal way to expose the thyroid and decided to utilize a head turn, thus maximizing the acoustic window. The subjects were asked to turn their head left when we were measuring on right side and vice versa (see Fig 1(A)). This procedure was monitored by a high frequency ultrasound transducer, which, due to its high spatial resolution, allowed to identify the different tissue structures. Two representative ultrasound images are shown in Fig 1(A)). The final locations according to Fig 1(A) and 1(C) were chosen depending on the physiology and identification by ultrasound prior to the diffuse optical measurements.

The non-invasive measurements employed diffuse light for two source-detector separations (13 and 25 mm) to probe the tissue properties and the necessary sources and detectors were embedded in a 4 cm x 4 cm soft black foam pad (see Fig 1(D)), which was placed directly on the subject’s skin. The shorter source-detector separation (SD) was used in a subset of eleven subjects, which is half of the study population, as well as the two pathology cases. The details are shown in Fig 1.

The static optical properties (absorption and reduced scattering) were measured with time-resolved spectroscopy (TRS). The computed absorption properties for each wavelength were used to calculate oxy- and deoxy-hemoglobin concentrations using extinction coefficients from the literature [33]. The blood flow index was monitored by diffuse correlation spectroscopy (DCS). The variations in the correlation decay times from the recorded intensity fluctuations translate into a parameter called “blood flow index”, which is directly related to microvascular blood flow.

One data set consisted of DCS data acquired for both source-detector separations (13 and 25 mm) simultaneously and TRS curves recorded individually for all wavelengths and source-detector separations with a acquisition time per individual curve of seven seconds (t^TRS^_25mm_ = 21s; t^TRS^_13mm_ = 21s; t^DCS^ = 7s). The experimental setup switched automatically between wavelengths and modalities. At each location, we have recorded three complete data sets leading to a total acquisition time of ⁓ 147 seconds per measurement location.

After one complete scan of the subject’s neck—according to the points marked in Fig 1(A) and 1(C)—a second identical scan followed. Low signal-to-noise ratio data was manually identified and excluded individually after the acquisition.

All measurements on nodule cases were performed at least 48 days after the FNAB, which is considered to be enough time to avoid contamination of the DOS measurements [34].

### Diffuse optics device & probe

Time-resolved spectroscopy (TRS) and diffuse correlation spectroscopy (DCS) were combined in a single instrument. The TRS part, used for the determination of total hemoglobin concentration (THC) and oxygen saturation (StO_2_), consisted of pulsed lasers (repetition rate: 50 Mhz) at the following three wavelengths: 690, 785 and 830 nm (BHLP-700, Becker & Hickl GmbH, Berlin, Germany) with pulse widths of 400, 350 and 450 picoseconds, respectively. The laser light was sent to the tissue using a 10 m long graded index multimode fiber with a 90° fiber head and a core diameter of 62.5 *μ*m (NA = 0.275). The fiber length was chosen to provide an ample amount of flexibility to operate in the clinic. For light detection a fifty-four fiber bundle of the same type multimode fibers was used. The light was collected directly at the tissue surface and guided to a hybrid photo multiplier detector (HPM-100-50, Becker & Hickl GmbH, Berlin, Germany) which was connected to a single photon counting and a detector control card (SPC-130 and DCC-100, Becker & Hickl GmbH, Berlin, Germany). The time-correlated single photon counting (TCSPC) setup was controlled by a manufacturer-provided software (SPCM, Becker & Hickl GmbH, Berlin, Germany) which acquired the distribution time of flight curves built from the diffused photons collected from the tissue [35, 36].

The DCS modality used a single longitudinal mode laser as a source (DL785-120-SO, CrystaLaser, Reno, NV, USA) with a long coherence length (much longer than a typical photon path length). The DCS laser light was sent to the tissue through a multimode fiber with a core diameter of 200 *μ*m (NA = 0.22) and the corresponding diffused light was collected by a bundle of four single mode fibers, each with a core diameter of 5.6 *μ*m, and guided from the tissue to four single-photon counting avalanche photodiodes (Excelitas, Québec, Canada). The intensity autocorrelation functions were calculated in real-time by a digital correlator (Correlator.com, New Jersey, USA) separately from each avalanche photodiode. For details on the functional principles please refer to [19, 37].

All subjects were measured with a source-detector separation of 25 mm for both, TRS and DCS. Furthermore, for a subset we have introduced additional detectors for both modalities at a 13 mm distance from the source into our probe to explore whether there are any differences in superficial tissues. The probe is illustrated in Fig 1(D).

### Ultrasound device and protocol

In our study, grayscale ultrasound (US) scans were used with the neck in hyperextension both in neutral position and after right and left neck rotation using a real-time ultrasonographic scanner with a 7.5 MHz linear transducer connected to a M7 Mindray US machine (Shenzhen, China). All US examinations were performed by a radiologist and an endocrinologist with extensive experience in thyroid ultrasound (twelve and six years respectively). The US screening consisted of longitudinal and transverse scans from the skull base to the supraclavicular and suprasternal region to identify normal relevant vascular and muscular cervical structures and to evaluate the thyroid gland. As explained above, cases with thyroid nodules, diffuse thyroid disease and cervical adenopathies were excluded from the healthy population. Grayscale US was further utilized to evaluate the thyroid gland position, the common carotid arteries and the jugular veins. Similarly, the sternocleidomastoid muscles were identified bilaterally in neutral position and during right and left lateral rotation. This allowed the evaluation of the normal sternocleidomastoid muscle situation and its displacement over to the deep cervical structures during neck movements (see also Fig 1(A)). The distances between the superficial tissue layer and the anterior and posterior border of the muscle belly were recorded. The echo structure and echogenicity of the thyroid parenchyma were analyzed and the thyroid lobes were measured in three different dimensions: craneo-caudal, latero-lateral and antero-posterior. Furthermore, the distances between the superficial tissue layer and the anterior and posterior border of the thyroid gland were measured at the middle third of the right and of left lobe.

For each subject, various other ultrasound images were taken. These images corresponded to the locations for the diffuse optics measurements and provided informations about the depth and thickness of the thyroid and the muscle depending on the location. An example US image from the thyroid is shown in Fig 1(B).

### Data evaluation

#### Time-resolved spectroscopy data analysis

As explained in Section “Diffuse optics device & probe”, the time-resolved spectroscopy (TRS) setup acquired distribution time of flight curves for three different wavelengths (λ = 690, 785 and 830 nm). A convolution of each instrument response function with a solution for the diffusion approximation for the semi-infinite homogeneous medium [38] of the radiation transport equation was fitted to the corresponding curve, which yields the absorption (*μ*_a_) and reduced scattering coefficients (*μ*_s_′). Each curve was normalized and the above-mentioned solution fitted to a range from 80% of the peak value on the rising edge to 1% on the tail based on the diffusion approximation and the nature of absorption.

The measured absorption coefficients (*μ*_a_ (λ)) were used to calculate the oxy- and deoxy-hemoglobin concentrations, since they relate to the different tissue chromophore concentrations (c_i_) via the individual extinction coefficients (ϵ(λ)): *μ*_*a*_(λ) = $\sum_{i}^{n}\epsilon_{i}\left(\lambda \right)\;c_{i}$. In case of this study we have considered only three different chromophores which were the main contributions to the measured signals at the above-mentioned wavelengths. The molar absorption coefficients for oxy- and deoxyhemoglobin were retrieved from Ref. [33] and the corresponding ones for water from Ref. [39]. Assuming a negligible lipid contribution and a water concentration (cH_2_O) of 78% (Ref. [40]) we have obtained the total hemoglobin concentrations and oxygen saturations for each data set.

#### Diffuse correlation spectroscopy data analysis

Diffuse correlation spectroscopy (DCS) measurements are sensitive to the dynamics of the scatterers in tissues. By recording intensity fluctuations of coherent diffused light, DCS uses a photon diffusion model to evaluate the mean displacement of the scatterers. It turns out, that in tissues, this is dominated by moving red blood cells in the microvasculature allowing us to estimate the microvascular blood flow [19].

The movement of the red blood cells adds a phase shift to the photon path in each scattering event, which in consequence causes random fluctuations in the electric field and therefore in the intensity of the observed laser speckles. Calculating the autocorrelations from these fluctuations for different delay times yields information about the dynamics in the tissue. The solution of the correlation diffusion equation for the semi-infinite homogeneous medium was used to fit to the computed normalized electric field autocorrelation function as described in Ref. [19]. As mentioned above, DCS measures local microvascular blood flow which we present as a blood flow index (BFI) for the probed region.

We note that for each subject and each measurement location we have used the *μ*_a_ and *μ*_s_′ values from the TRS measurement in the DCS analysis.

### Statistical analysis

All values reported in Tables 1–6 are mean values averaged over all included healthy subjects plus minus the standard deviation. The absorption and scattering coefficients (*μ*_a_, *μ*_s_′), the total hemoglobin concentrations (THC), the oxygen saturations (StO_2_) and the blood flow indices (BFI) were tested for normality with the “Shapiro-Wilk” test [41] and cross-checked by quantile-quantile plots. Both tests were in agreement with each other. This normality test serves as a quality check and for the suitability of the methods used for further statistical analysis. The primary method was the linear mixed effects (LME) model. The entire statistical analysis was carried out in R [42]. In general, “p-values” less than 0.05 were considered statistically significant to reject the null hypothesis. Within R, we have used the “nortest” [41] and the “nLME” [43] packages for our evaluation. LME fitted models which did not differ significantly from the null model were rejected. Furthermore, LME fitted values are used to confirm the reported mean values. The differences between genders in the demographical, vital and tissue dimension related parameters were tested using a two-tailed t-test.

**Table 1:** Demographic parameters.

	Age [yrs]	Weight* [kg]	Height* [cm]	BMI [kg/m^2^]
**All**	**32 ± 5**	**69 ± 13**	**171 ± 8**	**23.5 ± 3.6**
*Females*	*31 ± 4*	*60 ± 11*	*164 ± 6*	*22.2 ± 4.5*
*Males*	*32 ± 5*	*77 ± 9*	*177 ± 5*	*24.6 ± 2.5*

The demographic parameters of the population given as mean ± their standard deviation.

* denotes a statistically significant difference between females and males (t-test, p < 0.05).

**Table 2:** Vital records.

	HR [bpm]	SaO_2_ [%]	BP_sys_ [mmHg]		BP_dia_ [mmHg]	
			before*	after*	before*	after
**All**	**73 ± 14**	**98 ± 1**	**126 ± 15**	**127 ± 13**	**75 ± 10**	**75 ± 9**
*Females*	*75 ± 13*	*98 ± 1*	*114 ± 9*	*119 ± 10*	*69 ± 9*	*70 ± 4*
*Males*	*71 ± 14*	*98 ± 1*	*133 ± 14*	*132 ± 12*	*79 ± 8*	*78 ± 11*

The table shows the distribution (mean ± standard deviation) of the vital records: heart rate (HR), arterial oxygen saturation (SaO_2_), systolic and diastolic blood pressure (BP_sys_, BP_dia_).

*denotes a statistical significant difference between females and males (t-test, p < 0.05).

**Table 3:** Tissue dimensions recorded by ultrasound.

	**Muscle**		
	**STT [mm]**	TT* [mm]	TTD* [mm]
**All**	**2.9 ± 0.8**	**7.5 ± 1.9**	**10.4 ± 2.2**
*Females*	*2.9 ± 0.8*	*6.8 ± 1.7*	*9.7 ± 1.9*
*Males*	*3.0 ± 0.8*	*8.1 ± 1.8*	*11.1 ± 2.2*
	**Thyroid**		
	STT* [mm]	TT* [mm]	TTD* [mm]
**All**	**7.6 ± 2.3**	**12.1 ± 4.1**	**19.7 ± 4.9**
*Females*	*6.5 ± 2.0*	*10.4 ± 2.8*	*16.9 ± 3.3*
*Males*	*8.7 ± 2.0*	*13.7 ± 4.5*	*22.4 ± 4.6*

Means and standard deviations of the tissue dimensions. The definition of these three parameters is illustrated in Fig 1(B). The distance from the probe-skin contact to the deeper end of the target tissue figures as the total tissue depth (TTD), whereas the tissue thickness (TT) is difference between the total tissue depth and the superficial tissue thickness (STT).

*denotes a statistical significant difference between females and males (t-test, p < 0.05).

**Table 4:** Absorption coefficients (*μ*_a_) from TRS data.

	*μ*_a, 690_ [cm^-1^]	*μ*_a, 785_ [cm^-1^]	*μ*_a, 830_ [cm^-1^]
**Muscle (right)**	0.22 ± 0.04	0.21 ± 0.03	0.23 ± 0.03
**Gland location 1 (right)**	0.30 ± 0.04	0.28 ± 0.04	0.32 ± 0.05
**Gland location 2 (right)**	0.27 ± 0.04	0.26 ± 0.03	0.30 ± 0.04
**Gland location 2 (left)**	0.27 ± 0.05	0.26 ± 0.04	0.30 ± 0.05
**Gland location 1 (left)**	0.28 ± 0.05	0.27 ± 0.04	0.30 ± 0.06
**Muscle (left)**	0.22 ± 0.04	0.21 ± 0.03	0.24 ± 0.04

Absorption coefficients *μ*_a_ (mean ± standard deviation) deducted from the three wavelength of the time-resolved spectroscopy data. The thyroid gland shows a significantly (p < 0.05) higher absorption than the muscle at all locations.

**Table 5:** Reduced scattering coefficients (*μ*_s_′) from TRS data.

	***μ*_s,_′_690_ [cm^-1^]**	***μ*_s,_′_785_ [cm^-1^]**	***μ*_s,_′_830_ [cm^-1^]**
**Muscle (right)**	9.1 ± 0.9	8.1 ± 1.0	7.3 ± 0.9
**Gland location 1 (right)**	9.2 ± 1.1	7.9 ± 1.3	7.4 ± 1.1
**Gland location 2 (right)**	8.6 ± 1.2	7.4 ± 1.3	6.9 ± 1.1
**Gland location 2 (left)**	8.1 ± 1.2	7.0 ± 1.3	6.5 ± 1.2
**Gland location 1 (left)**	8.8 ± 1.1	7.6 ± 1.3	7.2 ± 1.2
**Muscle (left)**	9.1 ± 0.9	8.0 ± 0.9	7.3 ± 0.9

Reduced scattering coefficients *μ*_s_′ (mean ± standard deviation) for all three wavelengths obtained by time-resolved spectroscopy. The recorded data reveals statistically significant (p < 0.05) differences between the muscle and the thyroid gland.

**Table 6:** Calculated hemodynamic parameters.

	**THC [*μ*M]**	**StO_2_ [%]**	**BFI [cm^2^/s] × 10^-9^**
**Muscle (right)**	100.9 ± 13.7	63.8 ± 3.8	8.1 ± 4.3
**Gland location 1 (right)**	144.8 ± 21.4	67.4 ± 3.1	13.7 ± 4.5
**Gland location 2 (right)**	134.7 ± 18.0	68.4 ± 2.7	14.7 ± 6.1
**Gland location 2 (left)**	131.9 ± 22.2	67.5 ± 2.2	16.0 ± 8.8
**Gland location 1 (left)**	136.5 ± 26.8	66.6 ± 3.0	15.4 ± 7.3
**Muscle (left)**	105.7 ± 17.8	65.9 ± 3.4	7.8 ± 1.8

Means and standard deviation for total hemoglobin concentration (THC), oxygen saturation (StO_2_) and blood flow index (BFI) are shown here for all six measurement locations according to the study protocol. Rather strong differences (p < 0.0001) can be observed in the THC and BFI between the thyroid and the muscle, whereas the StO_2_ concentrations show small to almost no variations. Please see the text for details.

The hemodynamic variables (THC, StO_2_ and BFI) were investigated using (LME) models [43] where the subject number was considered as a random effect. These LME models tested for differences between organs, i.e., muscle and thyroid, looked for influences by probe location on the thyroid (“gland location 1” and “gland location 2”, see Fig 1(A) and 1(C)) or between thyroid lobe sides and checked for dependencies on physiological parameters and tissue dimensions (see Fig 1(B)). We fitted each of the LME models with the parameter under investigation (organ, location, side, physiological parameter or tissue dimension) as a fixed effect. We, furthermore, have double checked all our statistically significant findings by a bootstrapping analysis. In the case of eleven healthy subjects that were measured with a second source-detector separation of 13 mm, we have used the same LME models to check for dependencies on organs, sides, locations and tissue dimensions for that additional data.

## Results

### Study population

Twenty-two subjects (ten females and twelve males) out of a total of twenty-eight screened ones were included in the analysis as the healthy group for this study. The data of three volunteers was excluded due to technical issues. One subject was diagnosed with thyroiditis, one subject was diagnosed with a benign and another one a malignant thyroid nodule after the initial screening. The latter two are reported here as two pathologic cases studies (1 female and 1 male).

The basic health screening confirmed the overall health condition of the subjects. The demographic parameters from the healthy study population, such as age, weight, height and body mass index (BMI) are presented in Table 1, while the vital records including heart rate (HR), arterial oxygen saturation (SaO_2_), the systolic and diastolic blood pressure (BP_sys_, BP_dia_) before and after the optical measurement are shown in Table 2.

The thicknesses of the superficial tissues, the tissue of interest and the total tissue depth as measured by ultrasound are shown in Table 3 and are illustrated in Fig 1(B). The whole distance from the skin surface to the deeper border of the target tissue is considered as the total tissue depth (TTD), whereas the tissue thickness (TT) is defined by the difference between the total tissue depth and the corresponding superficial tissue thickness (STT).

### Optical and hemodynamic properties of the healthy population

On every measurement location, see Fig 1(A) and 1(C), we have obtained the absorption (*μ*_a_) and reduced scattering coefficients (*μ*_s_′) at three wavelengths employing time-resolved spectroscopy (TRS) as described in Sections “Diffuse optics device & probe” and “Time-resolved spectroscopy data analysis”. A representation of these values is shown in Tables 4 and 5. These mean values and their standard deviations for each location present the healthy population average. Three percent of the initial data was excluded due to a low signal-to-noise ratio.

In order to relate our optical measurements to the tissue hemodynamics, we have assumed the lipid contribution to be negligible and a water content of 78% [40], which led to total hemoglobin concentrations (THC), oxygen saturations (StO_2_) and blood flow indices (BFI) as shown in Table 6.

On the right neck side, the THC increases from 100.9 ± 13.7 *μ*M in the muscle to 134.7 ± 18.0 *μ*M (“gland location 2 (right)”) and 144.8 ± 21.4 *μ*M (“gland location 1 (right)”) in the thyroid lobe and on the left side from 105.7 ± 17.8 *μ*M in the muscle to 131.9 ± 22.2 *μ*M (“gland location 2 (left)”) and 136.5 ± 26.8 *μ*M (“gland location 1 (left)”), which represents a 33–44% increase on the right side and a 25–29% increase on the left side.

The BFI changes from 8.1 ± 4.3 cm^2^/s × 10^-9^ in the right muscle to 13.7 ± 4.5 cm^2^/s × 10^-9^ (“gland location 1 (right)”) and 14.7 ± 6.1 cm^2^/s × 10^-9^ (“gland location 2 (right)”) in the right thyroid lobe and on the left side from 7.8 ± 1.8 cm^2^/s × 10^-9^ in muscle to 15.4 ± 7.3 cm^2^/s × 10^-9^ (“gland location 1 (left)”) and 16.0 ± 8.8 cm^2^/s × 10^-9^ (“gland location 2 (left)”). This is a 70–81% blood flow increase from muscle to thyroid on the right neck side and a 97–105% higher blood flow in the left thyroid lobe than the respective muscle.

The StO_2_ values increase from 63.8 ± 3.8% in the muscle to 67.4 ± 3.1% (“gland location 1 (right)”) and 68.4 ± 2.7% (“gland location 2 (right)”) in the thyroid on the right side and on the left side from 65.9 ± 3.4% in the muscle to 66.6 ± 3.0% (“gland location 1 (left)”) and 67.5 ± 2.2% (“gland location 2 (left)”) in the thyroid region, which represents a change within the standard deviations.

A full subject-wise representation of the *μ*_a_, *μ*_s_′, THC, StO_2_ and BFI values can be found in the Tables in S1, S2 and S3 Tables.

#### Normality tests

As described in Section “Statistical analysis”, all variables (*μ*_a_, *μ*_s_′, THC, StO_2_ and BFI) have been checked for normality using the “Shapiro-Wilk” test [41] and cross-checked by quantile-quantile plots. According to these tests, the BFI values recorded in “gland location 2 (right and left)”, as well as in the “neck muscle (left)” location are not normally distributed (p ≤ 0.01).

#### Organ differences

From the corresponding LME model with the organ as a fixed effect, we have found a statistically significant (p < 0.0001) increase over all three wavelengths in the recorded absorption coefficient (*μ*_a_) at the thyroid locations with respect to the sternocleidomastoid muscle. The average *μ*_a_ from the thyroid are: *μ*_a, 690_ = 0.28 cm^-1^; *μ*_a, 785_ = 0.27 cm^-1^; *μ*_a, 830_ = 0.31 cm^-1^. In the muscle region we have recorded the following averaged absorption coefficients (*μ*_a_): *μ*_a, 690_ = 0.22 cm^-1^; *μ*_a, 785_ = 0.21 cm^-1^; *μ*_a, 830_ = 0.24 cm^-1^.

The same LME model employed on the scattering characteristics, i.e., the reduced scattering coefficients (*μ*_s_′), leads to statistically significant (p_690_ < 0.01; p_785_ < 0.001; p_830_ < 0.05) differences between organs. In the thyroid the reduced scattering coefficient (*μ*_s_′) values are *μ*_s,_′_690_ = 8.7 cm^-1^; *μ*_s,_′_785_ = 7.5 cm^-1^; *μ*_s,_′_830_ = 7.0 cm^-1^ and the ones for the muscle are *μ*_s,_′_690_ = 9.1 cm^-1^; *μ*_s,_′_785_ = 8.1 cm^-1^; *μ*_s,_′_830_ = 7.3 cm^-1^.

From the LME model with the organ as a fixed effect we find statistically significant (p < 0.0001) differences for THC, StO_2_ and BFI with the average thyroid tissue values of: THC = 137.1 *μ*M; StO_2_ = 67.5% and BFI = 15.0 × 10^-9^ cm^2^/s. For the muscle tissue we get: THC = 103.3 *μ*M; StO_2_ = 64.9% and BFI = 7.9 × 10^-9^ cm^2^/s.

#### Side and probe location differences

We have used LME models for further investigation of possible significant differences in the measured values between sides and thyroid probe locations.

In terms of left-right symmetry, we see that StO_2_ differs (p = 0.01) between the right and the left muscle in contrast to the thyroid glands, where we found no significant variations in none of the recorded values between the opposed sides. Here we have set the side information as a fixed effect and retrieve a fitted value for StO_2_ in the left sternocleidomastoid muscle of 65.9% and on the right side an oxygen saturation of 63.8%.

Apart from the StO_2_ in the muscle, a LME model with the probe location on the thyroid as a fixed effect fitted values which are statistically significantly different from the zero model for THC (p < 0.01) and StO_2_ (p = 0.02). In the gland location 2 THC and StO_2_ are 133.3 *μ*M and 68.0%, while in gland location 1 they are 125.4 *μ*M and 67.2%.

#### Age, gender and BMI influences

Furthermore, we have investigated influences from the subject demographics, such as body mass index (BMI), age and gender, which revealed an statistically significant dependence on BMI (p < 0.0001) for all hemodynamic parameters (THC, StO_2_ and BFI) and a significant influence of THC by age (p < 0.01) and gender (p = 0.03). THC, StO_2_, and BFI values decrease with higher BMI, as does THC in older subjects. For female subjects THC is in average 128.2 *μ*M and for the male subjects 144.7 *μ*M.

#### Dependencies on tissue dimensions

Additionally, we have looked into possible dependencies of THC, StO_2_ and BFI on the different tissue thicknesses (superficial tissue thickness and tissue thickness) and the total tissue depth (see Fig 1) and have found a significant decrease in THC with increasing superficial tissue thickness (p < 0.01).

#### Short (13 mm) source-detector separation

We have also recorded data for an additional source-detector separation (SD) of 13 mm from a subset of eleven healthy subjects (six females, five males) out of the twenty-two in total. The information from the shorter SD of 13 mm revealed different values and dependencies than the ones from a SD of 25 mm.

We performed the same statistical tests for organs, sides, locations and the three tissue dimension parameters (superficial tissue thickness, tissue thickness and total tissue depth) on that data subset with this short source-detector separation as before. Similar to the long SD data, these tests result in significant organ differences (p < 0.01) with lower THC, StO_2_ and BFI in the muscle locations.

StO_2_ shows not only a significant influence by side (p_muscle_ < 0.01) in the muscle location as for the SD of 25 mm, but also depends significantly on the thyroid lobe side (p_gland_ < 0.01), showing an increase on the left side. It is also lower with increasing STT (p < 0.01) and increases with larger TTD (p < 0.01). Also, in this source-detector configuration BFI is increasing with larger TTD (p < 0.01) and THC is smaller for larger STT (p < 0.01).

Furthermore, all hemodynamic variables are still significantly depending on BMI (p < 0.01), similar to the large source-detector separation data.

As before, a full subject-wise representation for this data subset of 11 subjects for *μ*_a_, *μ*_s_′, THC, StO_2_ and BFI values can be found in the Tables in S1, S2 and S3 Tables.

### Case studies with pathologies

**Fig 2:**
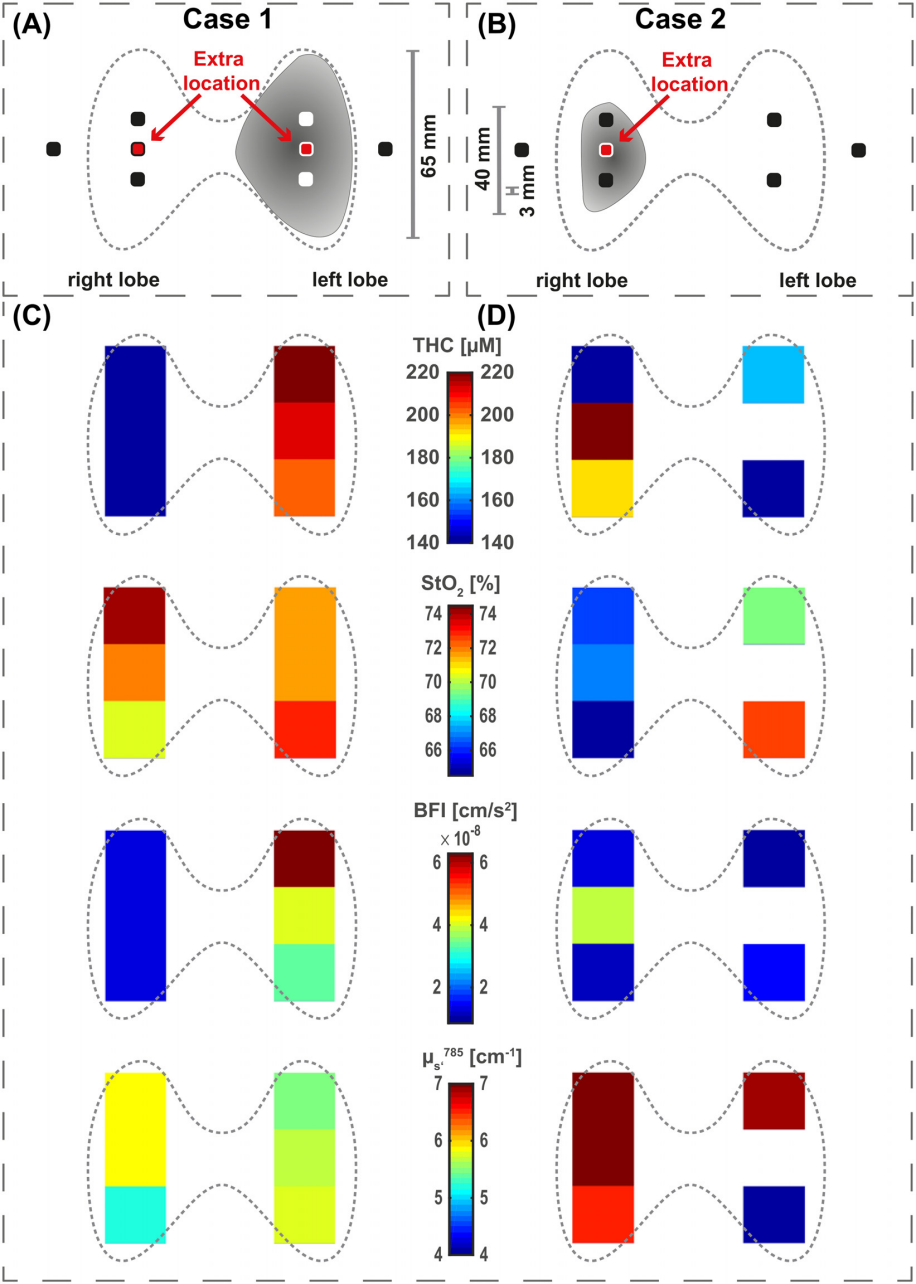
Thyroid schematics and results for the nodule cases. (A) *CASE 1*: Two locations were added to the standard protocol. The nodule was located within the left thyroid gland with a maximum diameter of 65 mm, as indicated by the shaded region. (B) *CASE 1*: One location was added on the right lobe to the standard protocol. This case had a maximum diameter 40 mm nodule (shaded region) together with a second nodule of 3 mm diameter, both located in the right thyroid gland. The results ((C) and (D)) are shown for the total hemoglobin concentration (THC), oxygen saturation (StO_2_), blood flow index (BFI) and the reduced scattering coefficient (*μ*_s_′) for 785 nm. Note, that these color plots do not include the muscle locations. For the detailed representation please refer to the Tables in S4 Table and in S5 Table.

In addition to the healthy population, we present two case studies that were previously diagnosed with a pathology. Fig 2(A) and 2(B) show the corresponding thyroid schematics for each case. The shaded regions represent the locations of the thyroid nodules and their approximate size in relation to the complete thyroid. The measurements consisted of the same procedure as for the healthy volunteers, explained in Section “Protocol” but with added measurement locations on the nodule side, i.e. “gland center right” (case 1 and 2) and “gland center left” (case 1).

#### Pathology case 1 (*CASE 1*)

The first case was a male (38 years old; weight 71 kg; height 1.64 m; BMI 26.4 kg/m^2^) with a thyroid nodule (maximum diameter > 65 mm) affecting the left lobe and extending into the isthmus. The nodule was heterogeneous with necrotic areas. The FNAB examination showed atypical cells suggestive of papillary thyroid cancer and MRI showed a destructured mass occupying the left thyroid lobe and the isthmus, infiltrating all surrounding thyroid structures (trachea and esophagus) and the skin. Due to the results, the tumor was considered unresectable and radio- and chemo-therapy with tyrosine kinase inhibitors (“Sorafenib”) was suggested as a neoadjuvant therapy prior to surgery.

The hybrid diffuse optics probe was applied on a total of eight locations in this case (see Fig 2(A)). The extra locations were added in order to ensure that nodule is measured. The hybrid diffuse optics results are shown in Fig 2(C). We see that in all three locations on the left lobe the total hemoglobin concentrations (THC) and blood flow indices (BFI) are higher than the right thyroid side (p < 0.0001). We have defined a nodule index which was set positive in all probe location on the left thyroid gland—as opposed to the nodule-free right thyroid gland where that index was set to zero—and fitted a LME model with the nodule index as a fixed effect. The right thyroid lobe values average to THC = 138.3 *μ*M and BFI = 12.8 × 10^-9^ cm^2^/s, and, on the nodule, in the left thyroid lobe they increase to THC = 210.8 *μ*M and BFI = 45.5 × 10^-9^ cm^2^/s. Additionally, the values show a higher variability with three to nine times higher standard deviations especially in the gland location 1 on the nodule side and are distinguishable from the contralateral lobe. The observed standard deviations were: *σ*_THC, left_ = 3.7–11.4 *μ*M versus *σ*_THC, right_ = 1.2–2.4 *μ*M; *σ*_StO_2_, left_ = 0.8–3.3% versus *σ*_StO_2_, right_ = 0.5–2.8%; *σ*_BFI, left_ = (3.0–6.4) × 10^-9^ cm^2^/s versus *σ*_BFI, right_ = (0.5–1.2) × 10^-9^ cm^2^/s. Moreover, we observe a relatively high oxygen saturation (StO_2_) throughout the whole thyroid (StO_2_ = 70.2–74.1% compared to StO_2, healthy_ = 66.6–68.4%, while the reduced scattering coefficient (*μ*_s_′) in the thyroid turns out lower than the healthy population (*μ*_s_′ = 5.2–5.7 cm^-1^ compared to *μ*_s_′_, healthy_ = 7.0–7.9 cm^-1^). A tabular representation of this case can be found in S4 Table.

#### Pathology case 2 (*CASE 2*)

As a second case we present results from a female (32 years old; weight 68 kg; height 1.82 m; BMI 20.5 kg/m^2^) with a a thyroid nodule (40 mm) in the right lobe with foci of colloid degradation and peripheral vascularisation suggestive of a hyperplastic nodule. An additional small nodule of 3 mm diameter in the right lobe was observed, with no laterocervical adenopathies. Fine needle aspiration biopsy was performed and was suggestive of a follicular lesion. Furthermore, the nodule showed microfollicular characteristics and was classified according to the “Bethesda system for reporting thyroid cytopathology” [44] which scales from one to six with increasing malignancy risk as Bethesda 4. On this subject, after the optical measurements, a total thyroidectomy was performed and histopathology showed multinodular thyroid hyperplasia with a hyperplastic dominant nodule (maximum diameter of 4 cm) and regressive changes with adenomatoid nodules. No follicular neoplasms were observed. Finally, the pathology was classified as benign.

Fig 2(D) shows the diffuse optics results. Similar to the previous case, a nodule index was defined and set to positive in “gland location 2” and “gland center”. With the same LME model as for *CASE 1* we have found a significant difference from the null model (p < 0.01) with a THC for a positive nodule index of 206.8 *μ*M in contrast to 147.9 *μ*M for non-nodule locations. The other parameters (StO_2_, BFI and *μ*_s_′) are not significantly influenced by the two nodule locations. The *μ*_s_′ values in three thyroid locations are lower than the ones retrieved from the healthy population (*μ*_s_′ = 4.0–6.5 cm^-1^ against *μ*_s_′_, healthy_ = 7.0–7.9 cm^-1^). A tabular representation of this case can be found in S5 Table.

## Discussion

After a general reflection of this study’s achievements, we separate the discussion into subsections focusing on various aspects in a detailed manner.

### Overview

The first result is that the proposed protocol including the positioning of the subject, the neck and the diffuse optics probe placement locations were sufficiently optimized. The measurement protocol that was drawn-up (see Section “Protocol”) allowed a good signal-to-noise-ratio and a good fitting quality for TRS and DCS data sets as well as an appropriate and convenient total measurement duration per subject (30–45 min). At the end, the optical and hemodynamic parameters of the human thyroid and the sternocleidomastoid muscle tissue from twenty-two healthy (ten females, twelve males) and two subjects with a thyroid pathology were investigated. The overall procedure demonstrates that it is possible to relate the diffuse optical signal to the hemodynamics in the target tissue.

The measurements appear to be robust in terms of repeatability since there was no perceivable difference between values from the same subject obtained at different times, neither over the time span of the measurement (⁓ 30 minutes) nor for a time span of 112 months (data not shown). For the latter, we rely on data from a subject who has volunteered twice within 112 months and during which we have conducted several probe placements per location. The variations were within the obtained standard deviations. Furthermore, we note that male beard growth in the region did not pose any specific influence on our signal, whereas turning the subject’s head in order expose the thyroid—as verified by ultrasound—showed a clear positive effect (see also Fig 1(A)).

Varying the assumed water content ± 5% around the 78% we used in the analysis did not show a noticeable influence and since thyroid and neck muscle tissue are supposed to have a similar water concentrations [40] we did not assume different concentrations for each tissue. In the future, one could expand the wavelength range to determine water and lipid concentrations [45].

The normality tests on the optical and hemodynamic parameters (*μ*_a_, *μ*_s_′, THC StO_2_ and BFI) resulted in a non-normality for BFI in three locations (“gland location 2 (right and left)” and “neck muscle (left)”). This was observed in previously reported absolute BFI values [32] and may correctly reflect the underlying physiology or may be related to the technique. Moreover, we would like to point out, that while the majority of DCS results reported so far represent relative blood flow changes with respect to a time or tissue reference and the correct estimation of BFI depends on the optical tissue characteristics [19, 27, 46, 47], we have used TRS data in the DCS analysis leading to absolute, more accurate BFI values.

The probe locations were identified by an experienced radiologist and for each location and subject an ultrasound image was taken. Based on these images, we deduce, that the probed region by the diffuse optics device extends below the superficial tissues into the target tissues (muscle and thyroid) and not into the further underlying regions.

The main target tissue was the thyroid gland and its homogeneity which we have investigated by including two probe locations on each lobe gland. We have also measured the nearby muscles as a comparison. The overall set of locations was exactly the same on both sides of the human neck.

The computed values for the total hemoglobin concentration (THC), oxygen saturation (StO_2_) and the blood flow, represented by the blood flow index (BFI), indicate high levels of vascularisation and blood flow (Table 6), which is in accordance to previous measurements conducted by arterial spin labeling magnetic resonance imaging [48].

### The extent of the probed regions

Using ultrasound guidance it is possible to identify different anatomical structures in the neck region. High frequency ultrasound, with frequencies between 7–13 Mhz, and the associated high spatial resolution permits to identify the thyroid gland, the trachea and the esophagus in the anterior compartment of the neck, the common carotid artery and the jugular vein in the lateral compartment of the neck as well as the muscular structures both in the central and lateral compartment of the neck. When using ultrasound guidance it is therefore possible to mark the anatomical landmarks of the thyroid and of the other anatomical structures of the neck on the skin and to identify the position of diffuse optics probe to correctly analyze different anatomical structures without their superposition, thus minimizing interferences in the optical analysis. The mentioned slight head tilt and turn implemented in the protocol assisted this minimization further. Representative ultrasound images of this procedure and its effect are shown in Fig 1(A).

The results from the larger source-detector separation, which presumably probes deeper regions of the tissue, showed a significant (p < 0.0001) difference between the two organs (muscle vs thyroid) for all parameters. This implies that we are in fact probing the tissues underneath the superficial layers.

To further investigate this point, we have studied the dependence of THC, StO_2_ and BFI on the three variables defining the tissue dimensions: superficial tissue thickness (STT), total tissue depth (TTD) and tissue thickness (TT) (according to Fig 1(B)). STT appears to have an influence on the calculated THC in the thyroid lobes (p < 0.01). In a bootstrap analysis we have found out that by exclusion of only one subject (ID: 19) the statistical significant dependence of THC on superficial tissue thickness disappears (p = 0.08). This strengthens our assumptions that the majority of the detected photons carry information about the tissue of interest. The fact that subject 19 is the one with the lowest BMI (18.3 kg/m^2^) in our study population taken together with the general influence of BMI (p < 0.01) on the data explains the found effect in the bootstrapping analysis.

For further support to the claim that we are probing the thyroid gland, we note that different dependencies on the tissue dimensions were observed from the shorter SD of 13 mm, which has presumably probed shallower regions and was incorporated into our probe for a subset of eleven subjects (six females, five males). The THC and StO_2_ values in this case are dependent on the thickness of the superficial layers (p < 0.01), i.e. the superficial tissue thickness, thus indicating that this shorter separation was in fact probing a more superficial region. Additionally, there were no significant differences between gland location 1 and 2 in this configuration.

In summary, when looking at the dependence on the three above-mentioned variables for the tissue dimensions, the results indicate that we reach the thyroid tissue and the signal is dominated by that tissue. Nonetheless, we can not entirely rule out possible contamination from other tissues in some subjects, such as the jugular vein and /or the carotid artery. We have done our best to develop a protocol to specifically address this potential problem, including the neck extension for thyroid exposure, as explained in Section “Protocol” and Fig 1.

### Lobe (Side) differences

Apart from a significant difference for oxygen saturation (StO_2_) in the muscle location, we did not find any side influence on the values. In general, side differences in healthy subjects would be surprising, which is why we suspect the existence of the mentioned StO_2_ muscle side variation lies in the systematic way we placed our probe on the different locations and/or is produced by a single subject. We now discuss this further.

In the measurements, we have always started from the muscle location on the right side moving point by point to the left and then repeated the whole procedure. Since the subject was asked to slightly turn the head according to each probe location, the muscle in the last position was probably less relaxed as the one in the starting position. This may explain the slightly higher values—indicating a higher active region—for the left neck muscle location and the significant influence. The fact that the values from the muscle in the short SD, which is more sensitive to that region, also showed side influences further supports that assumption.

Furthermore, a bootstrap analysis revealed that the significant difference between muscle sides in StO_2_ for the SD of 25 mm (p = 0.01) becomes insignificant p = 0.06 by excluding subjects 12 and 18. This strong influence by only two subjects can be attributed to the way of recording our data, as explained above and a slight protocol modification can account for this effect. Nevertheless, this finding does not change our main hypothesis that we are able to probe the thyroid gland.

As mentioned above, the thyroid values from different sides were not significantly different (Section “Optical and hemodynamic properties of the healthy population”), which is expected. At the same time, our results indicated a dependence on probe location for THC (p < 0.01) and StO_2_ (p = 0.02) between gland location 1 and gland location 2. Although, we tried to maximally expose the thyroid by a head tilt and turn, we could see in the ultrasound images that we could not achieve this by 100%. Due to the dimensions of the thyroid and the two probe locations on the thyroid as defined in our protocol, we may probe the thyroid better in one location than the other, therefore leading to a change in the computed values.

### Influences of the subject demographics

We have found further influences on our data by body mass index (BMI) (p < 0.0001), age (p_THC, age_ < 0.01) and gender (p_THC, gender_ = 0.03). Similar to other influences (Section “The extent of the probed regions”) bootstrapping revealed a rather strong influence by one (female) subject (ID: 7). Its exclusion leads to a statistically non-significant gender relation (p_THC, gender_ = 0.07) and to a disappearance of age influence on THC (p_THC, age_ > 0.05). Nonetheless, the BMI influence remains significant, which seems reasonable. That diffuse optical measurements are sensitive to BMI has been shown previously in human breast tissue [49–51] and bone marrow [32]. Since diffuse optics is a non-invasive technique, one always has to deal with superficial tissues such as skin and adipose layers, which in turn make the optical measurements influenceable by BMI. Furthermore, there are indications that the BMI can actually influence the thyroid function [52, 53], which can partially explain the significant BMI dependence in our data. In the future, a multi source-detector probe can investigate this further.

We note that while these bootstrapping analyses have helped us to resolve unexpected results, they also indicate that future studies with a larger subject population are important to further investigate any dependencies on subject demographics, anatomy and physiology. This current study serves as the basis for this and establishes the variability of the results, which is needed for power calculations.

### Pathology cases

The case studies introduced in Section “Case studies with pathologies” show side differences between the glands and clearly higher vascularisation as indicated by elevated THC and BFI values compared to the healthy population. Assuming that the pathologic nodules are in the thyroid gland below the superficial regions, this further proves that we are sensitive to the thyroid’s microvasculature and that we are able to resolve differences in the thyroid glands. The data from *CASE 2* even suggests differences within the same gland, which is noteworthy considering the typical thyroid dimensions and the spatial resolution from diffuse optics. Furthermore, we see higher standard variations in the nodule signals in both pathologic cases, which may be due to the abnormal, unstable hemodynamics of the pathologic tissue volumes.

In *CASE 1*, we use THC (p_THC_ < 0.0001) and blood flow (p_BFI_ < 0.0001) to differentiate the lobe with the 65 mm nodule from the nodule-free side, which according to the FNAB results was suggestive of papillary thyroid carcinoma. This is in accordance to previous studies which have shown an increased microvasculature in papillary thyroid carcinoma [9].

As for *CASE 2* and its 40 mm nodule in the right lobe, we have observed a significant (p_THC_ < 0.01) THC increase in the lobe with the nodule. Furthermore, we note higher blood flows in the nodule, in particular from the added location (“gland center right”). The fact that we do not record higher values in all probe locations from the infected lobe are due to the smaller nodule dimensions compared to *CASE 1*.

In *CASE 1*, the oxygen saturations (StO_2_) throughout the whole thyroid are noticeably higher than in the healthy population and allow for no distinction between the presumably healthy and the nodule side. This is similar to the reduced scattering coefficients (*μ*_s_′) which are similar within both thyroid lobes and are relatively low compared to the healthy population.

For *CASE 2*, StO_2_ differs between thyroid lobes and tends to be lower on the nodule side, which might be explained by an increased oxygen consumption due to the nodule. This different behavior between the two cases, with a StO_2_ increase in *CASE 1* and a decrease in *CASE 2*, makes it difficult to use this variable as a discriminator at this point. It seems likely that *μ*_s_′ could be helpful in the search for a contrast between tumor types, since the malignant case presents values on the lower end of the scale, while the benign case lies somewhat in between the healthy population and malignant nodule. This is supported by previous results from a study by Suh et. al. [16] conducted on twenty patients. They have found that elastic scattering spectroscopy (ESS) can help discriminate between benign and malignant thyroid nodules due to its sensitivity to morphologic characteristics. Similar to ESS, DOS is sensitive to index of refraction changes, i.e., changes in nuclear size and density. These cellular arrangements were confirmed to change among different thyroid nodule types by Hung et al. [18] using two-photon excited fluorescence (TPEF) together with second-harmonic generation (SHG). Altogether results from ESS and TPEF with SHG support our findings and encourage the use of *μ*_s_′ as a further parameter to improve the thyroid screening process.

Apart from changes in the scattering characteristics, higher vascularisation in nodules is expected [28, 54]. The thyroid, in particular, as an already highly vascularised organ is showing even higher degrees of vascularisation when a nodule is developed, thus leading to increase hemoglobin concentrations and blood flows [48, 55]. This can be even more present on the microvascular level [9]. Our findings are in agreement with this on both the general as well as microvascular level and are therefore a promising approach towards an improvement of the thyroid screening procedure.

### Conclusion

The goal of this study was to establish a normal range for optical parameters of the healthy thyroid and their possible influences by physiological parameters, so that in the end these measurements can be compared to values obtained in the same way from pathologic thyroid tissues. We have shown that it is feasible to do diffuse optical measurements on the thyroid in-vivo and that the signal we are receiving is giving information about the thyroid vascularisation. This is supported by the different tests on dependencies of tissue dimensions and hemodynamic parameters (THC, StO_2_, BFI) as well as the contrast between healthy and pathologic tissue.

As expected, the two thyroid lobes do not show any significant difference in healthy subjects, whereas the signal from subjects with thyroid nodules clearly differed between sides. The observed dependence on the probe location demonstrates that it is important to use various probe placements on the gland. The fact that we did not record any changes over time (30 min to 1 12 months) in the healthy thyroid tissue demonstrates the robustness and repeatability of such a protocol.

The signals from nodule locations result in elevated microvascular THC values, which makes this a promising parameter for improvement in thyroid screening. There are further indications for higher BFI and altered *μ*_s_′ from the pathologic data encouraging the use of diffuse optics on thyroid tissue.

Finally, we can say, that our observations prove that our signal is in fact strongly influenced by the thyroid tissue, that we are able to characterize differences in the nature of this tissue and therefore paving the way of an improvement in sensitivity and specificity in thyroid screening by diffuse optical methods.

As a next step, we will employ diffuse optics on further thyroid nodule cases and other pathologies in order to improve thyroid screening methods and to compare healthy and pathologic distributions.

## Supporting Information

S1 Table Muscle location data. Means and standard deviations as a result of a total of six acquisitions taken in this location recorded with a source detector separation of 25 mm for the entire healthy population (n = 22) and one of 13 mm for a subset (n = 11) of the study population. Shown are the absorption coefficients (*μ*_a_) and reduced scattering coefficients (*μ*_s_′) per wavelength as well as total hemoglobin concentrations (THC), oxygen saturations (StO_2_) and blood flow indices (BFI).
(PDF)

S2 Table Gland location 1 data. Means and standard deviations as a result of a total of six acquisitions taken in this location recorded with a source detector separation of 25 mm for the entire healthy population (n = 22) and one of 13 mm for a subset (n = 11) of the study population. Shown are the absorption coefficients (*μ*_a_) and reduced scattering coefficients (*μ*_s_′) per wavelength as well as total hemoglobin concentrations (THC), oxygen saturations (StO_2_) and blood flow indices (BFI).
(PDF)

S3 Table Gland location 2 data. Means and standard deviations as a result of a total of six acquisitions taken in this location recorded with a source detector separation of 25 mm for the entire healthy population (n = 22) and one of 13 mm for a subset (n = 11) of the study population. Shown are the absorption coefficients (*μ*_a_) and reduced scattering coefficients (*μ*_s_′) per wavelength as well as total hemoglobin concentrations (THC), oxygen saturations (StO_2_) and blood flow indices (BFI).
(PDF)

S4 Table Pathology case 1 (*CASE 1*). Total hemoglobin concentration (THC), oxygen saturation (StO_2_), blood flow index (BFI) and the reduced scattering coefficient (*μ*_s_′) for all eight measurement locations according to the study protocol for this patient. Values are means and according standard deviations of several probe placements. * denotes the nodule locations.
(PDF)

S5 Table Pathology case 2 (*CASE 2*). Total hemoglobin concentration (THC), oxygen saturation (StO_2_), blood flow index (BFI) and the reduced scattering coefficient (*μ*_s_′) for all eight measurement locations according to the study protocol for this patient. Values are means and according standard deviations of several probe placements. * denotes the nodule locations.
(PDF)
