# Supplementary material for: Diffuse Optical Characterization of the Healthy Human Thyroid Tissue and Two Pathological Case Studies
Source: PLoS One. 2016 Jan 27;11(1):e0147851. doi: 10.1371/journal.pone.0147851 (PMC4731400; doi:10.1371/journal.pone.0147851)
Supplement: S1 Table — Means and standard deviations as a result of a total of six acquisitions taken in this location recorded with a source detector separation of 25 mm for the entire healthy population (n = 22) and one of 13 mm for a subset (n = 11) of the study population. Shown are the absorption coefficients (μa) and reduced scattering coefficients (μs′) per wavelength as well as total hemoglobin concentrations (THC), oxygen saturations (StO2) and blood flow indices (BFI). (PDF) [file pone.0147851.s001.pdf]

Table 1: **S1 Table. Muscle location data.**

ID	$\mu_{a,\,690}$ [cm^{-1}]	$\mu_{a,\,785}$ [cm^{-1}]	$\mu_{a,\,830}$ [cm^{-1}]	$\mu_{s,}{}'_{690}$	$\mu_{s,}{}'_{785}$	$\mu_{s,}{}'_{830}$	THC [μ M]	StO_2 [%]	BFI [cm^2/s] × 10^{-9}
**25 mm**									
1	0.25 ± 0.01	0.24 ± 0.01	0.27 ± 0.01	9.8 ± 0.2	9.0 ± 0.2	7.9 ± 0.2	119.6 ± 3.9	66.7 ± 1.4	10.7 ± 1.9
2	0.24 ± 0.01	0.20 ± 0.01	0.22 ± 0.01	9.0 ± 0.3	8.1 ± 0.2	7.4 ± 0.2	97.8 ± 3.0	57.9 ± 2.1	7.3 ± 1.4
3	0.24 ± 0.01	0.22 ± 0.01	0.24 ± 0.01	9.0 ± 0.6	8.2 ± 0.5	7.1 ± 0.4	109.5 ± 3.3	63.7 ± 1.9	7.1 ± 0.6
4	0.23 ± 0.01	0.21 ± 0.01	0.24 ± 0.01	9.0 ± 0.2	8.2 ± 0.1	7.6 ± 0.3	104.9 ± 5.3	64.8 ± 1.6	8.2 ± 1.0
5	0.22 ± 0.01	0.22 ± 0.01	0.24 ± 0.02	10.1 ± 0.2	9.1 ± 0.2	8.1 ± 0.2	107.1 ± 6.9	66.8 ± 1.9	13.4 ± 3.0
6	0.22 ± 0.02	0.23 ± 0.01	0.26 ± 0.01	8.8 ± 0.2	7.5 ± 0.2	6.7 ± 0.3	115.9 ± 5.1	70.0 ± 2.7	11.0 ± 1.3
7	0.19 ± 0.01	0.19 ± 0.01	0.20 ± 0.01	10.0 ± 0.2	9.4 ± 0.2	8.3 ± 0.2	88.4 ± 3.3	64.0 ± 1.4	5.8 ± 0.8
8	0.31 ± 0.03	0.26 ± 0.01	0.29 ± 0.01	7.9 ± 0.4	6.5 ± 0.3	5.8 ± 0.2	131.8 ± 5.4	60.4 ± 2.9	13.8 ± 2.3
9	0.21 ± 0.01	0.19 ± 0.01	0.21 ± 0.01	9.8 ± 0.3	8.6 ± 0.3	7.7 ± 0.2	92.7 ± 3.4	63.5 ± 1.2	11.2 ± 1.7
10	0.20 ± 0.01	0.19 ± 0.01	0.22 ± 0.01	7.4 ± 0.1	6.2 ± 0.1	5.7 ± 0.1	94.3 ± 2.0	66.6 ± 1.2	9.8 ± 1.5
11	0.18 ± 0.01	0.17 ± 0.01	0.19 ± 0.01	8.7 ± 0.2	7.5 ± 0.1	6.9 ± 0.2	80.2 ± 2.5	63.6 ± 1.9	6.0 ± 0.7
12	0.27 ± 0.01	0.26 ± 0.01	0.28 ± 0.01	11.5 ± 0.6	10.3 ± 0.5	9.6 ± 0.5	127.0 ± 3.2	64.7 ± 1.7	4.8 ± 1.1
13	0.23 ± 0.01	0.20 ± 0.01	0.22 ± 0.01	8.8 ± 0.1	7.3 ± 0.1	6.8 ± 0.1	97.9 ± 3.3	61.3 ± 1.8	9.2 ± 2.8
14	0.17 ± 0.01	0.17 ± 0.01	0.19 ± 0.01	8.7 ± 0.2	7.7 ± 0.2	7.1 ± 0.2	80.4 ± 2.8	66.8 ± 2.2	8.6 ± 1.5
15	0.26 ± 0.01	0.23 ± 0.01	0.26 ± 0.01	8.0 ± 0.1	7.2 ± 0.1	6.4 ± 0.1	115.2 ± 1.9	62.6 ± 1.2	6.6 ± 2.1
16	0.17 ± 0.01	0.19 ± 0.01	0.21 ± 0.01	8.4 ± 0.2	8.0 ± 0.2	7.1 ± 0.2	92.1 ± 6.6	70.8 ± 1.5	5.6 ± 1.2
17	0.20 ± 0.01	0.20 ± 0.01	0.22 ± 0.01	9.4 ± 0.1	8.2 ± 0.1	7.6 ± 0.1	98.6 ± 1.4	67.3 ± 2.1	7.0 ± 2.0
18	0.20 ± 0.01	0.18 ± 0.01	0.21 ± 0.01	8.9 ± 0.1	7.7 ± 0.1	7.3 ± 0.1	90.1 ± 3.1	62.6 ± 1.9	5.7 ± 0.6
19	0.23 ± 0.01	0.21 ± 0.01	0.23 ± 0.01	9.3 ± 0.3	8.2 ± 0.2	7.8 ± 0.2	104.0 ± 5.7	64.4 ± 1.6	4.3 ± 0.6
20	0.26 ± 0.01	0.24 ± 0.01	0.29 ± 0.01	8.9 ± 0.1	7.6 ± 0.1	7.5 ± 0.1	126.6 ± 3.0	67.6 ± 1.1	4.3 ± 0.4
21	0.21 ± 0.01	0.19 ± 0.01	0.21 ± 0.01	9.1 ± 0.2	8.1 ± 0.1	7.5 ± 0.1	94.0 ± 3.0	62.9 ± 1.4	5.9 ± 1.3
22	0.19 ± 0.01	0.19 ± 0.01	0.21 ± 0.01	9.8 ± 0.1	8.8 ± 0.1	8.1 ± 0.2	93.2 ± 3.4	67.7 ± 1.4	7.9 ± 1.6
**13 mm**									
3	0.21 ± 0.01	0.20 ± 0.01	0.20 ± 0.01	10.10 ± 1.2	9.5 ± 1.1	7.6 ± 1	92.3 ± 3.4	60.9 ± 2.3	9.4 ± 1.5
13	0.23 ± 0.01	0.21 ± 0.01	0.21 ± 0.01	13.20 ± 0.3	11.0 ± 0.2	9.9 ± 0.2	97.3 ± 4.5	60.0 ± 1.9	8.5 ± 3.2
14	0.17 ± 0.01	0.17 ± 0.01	0.18 ± 0.01	11.60 ± 0.2	10.1 ± 0.2	9.4 ± 0.2	77.8 ± 2.4	64.7 ± 2.9	7.8 ± 1.6
15	0.25 ± 0.01	0.23 ± 0.01	0.23 ± 0.01	10.60 ± 0.6	9.9 ± 0.5	8.1 ± 0.5	108.6 ± 2.7	60.4 ± 1.9	8.3 ± 2.7
16	0.16 ± 0.01	0.18 ± 0.01	0.19 ± 0.01	8.90 ± 0.2	8.9 ± 0.1	7.3 ± 0.2	84.9 ± 6.5	70.3 ± 1.0	5.6 ± 0.8
17	0.18 ± 0.01	0.17 ± 0.01	0.20 ± 0.01	10.90 ± 0.1	9.1 ± 0.2	8.8 ± 0.1	85.5 ± 1.9	65.4 ± 2.3	13.6 ± 1.9
18	0.19 ± 0.01	0.17 ± 0.01	0.19 ± 0.01	11.70 ± 0.3	9.6 ± 0.3	9.4 ± 0.3	81.7 ± 2.8	60.9 ± 2.6	7.3 ± 1.7
19	0.22 ± 0.01	0.19 ± 0.01	0.21 ± 0.01	12.40 ± 0.4	10.5 ± 0.3	10.4 ± 0.2	93.6 ± 4.4	61.5 ± 2.4	8.2 ± 1.4
20	0.26 ± 0.01	0.24 ± 0.01	0.27 ± 0.01	12.80 ± 0.3	10.5 ± 0.2	10.4 ± 0.2	122.2 ± 3.5	64.9 ± 1.1	8.6 ± 1.0
21	0.20 ± 0.01	0.18 ± 0.01	0.19 ± 0.01	11.20 ± 0.3	9.7 ± 0.3	8.9 ± 0.3	83.6 ± 3.4	60.1 ± 2.4	7.5 ± 1.1
22	0.21 ± 0.01	0.21 ± 0.01	0.22 ± 0.01	12.90 ± 0.4	11.1 ± 0.3	10.2 ± 0.3	99.9 ± 5.3	66.4 ± 1.1	8.7 ± 1.7

Means and standard deviations recorded with a source detector separation of 25 mm for the entire healthy population (n = 22) and one of 13 mm for a subset (n = 11) of the study population. Shown are the absorption coefficients μ_a and reduced scattering coefficients μ_s' per wavelength as well as total hemoglobin concentrations (THC), oxygen saturations (StO_2) and blood flow indices (BFI).
