# Supplementary material for: Diffuse Optical Characterization of the Healthy Human Thyroid Tissue and Two Pathological Case Studies
Source: PLoS One. 2016 Jan 27;11(1):e0147851. doi: 10.1371/journal.pone.0147851 (PMC4731400; doi:10.1371/journal.pone.0147851)
Supplement: S4 Table — Total hemoglobin concentration (THC), oxygen saturation (StO2), blood flow index (BFI) and the reduced scattering coefficient (μs′) for all eight measurement locations according to the study protocol for this patient. Values are means and according standard deviations of several probe placements. * denotes the nodule locations. (PDF) [file pone.0147851.s004.pdf]

Table 1: **S4 Table. Pathology case 1** (*CASE 1*).

	**THC [μM]**	**StO$_2$ [%]**	**BFI [cm^2/s] $\times$ 10^{-9}**	**μ_s,$'_{785}$ [cm^{-1}]**
**Muscle (right)**	82.6 ± 0.8	71.9 ± 1.2	7.2 ± 0.2	7.5 ± 0.1
**Gland location 1 (right)**	138.5 ± 1.6	74.1 ± 0.5	12.9 ± 1.2	5.9 ± 0.1
**Gland location 2 (right)**	134.7 ± 1.2	70.2 ± 0.5	12.9 ± 1.1	5.2 ± 0.1
**Gland location 2 (left)***	**201.3 ± 3.7**	**72.9 ± 2.2**	**33.1 ± 3.4**	**5.7 ± 0.3**
**Gland location 1 (left)***	**221.2 ± 11.4**	**71.6 ± 3.3**	**62.8 ± 6.4**	**5.5 ± 0.1**
**Muscle (left)**	92.7 ± 1.5	66.1 ± 3.3	17.9 ± 4.1	7.7 ± 0.3
**Gland center (right)**	141.8 ± 2.4	71.8 ± 2.8	12.5 ± 0.5	5.9 ± 0.2
**Gland center (left)***	**210.0 ± 6.1**	**71.6 ± 0.8**	**40.6 ± 3.0**	**5.7 ± 0.1**

Total hemoglobin concentration (THC), oxygen saturation (StO$_2$), blood flow index (BFI) and the reduced scattering coefficient ($\mu_s{}'$) for all eight measurement locations according to the study protocol for this patient. Values are means and according standard deviations of several probe placements. * denotes the nodule locations
